# DNA-Mimic Antirestriction Proteins ArdA Could Regulate Gene Expression in *Escherichia coli*

**DOI:** 10.3390/ijms27125595

**Published:** 2026-06-20

**Authors:** Anna A. Utkina, Anna A. Kudryavtseva, Rodion V. Berezov, Kamilla V. Mekhantseva, Olga E. Melkina, Sergey M. Rastorguev, Mikhail A. Skutel, Artem B. Isaev, Ilya V. Manukhov

**Affiliations:** 1 Moscow Center for Advanced Studies, 123592 Moscow, Russiamanukhovi@mail.ru (I.V.M.); 2 Kurchatov Complex of Genetic Research, NRC ‘Kurchatov Institute’, Kurchatov Square, 1, 123182 Moscow, Russia; 3 Pirogov Russian National Research Medical University, 117513 Moscow, Russia; 4 Center for Bio- and Medical Technologies, 121205 Moscow, Russiaa.isaev@skol.tech (A.B.I.)

**Keywords:** antirestriction, ArdB, ArdA, restriction, biosensor

## Abstract

Antirestriction proteins protect mobile genetic elements from the host’s restriction-modification systems. Here, we investigated the ability of ArdA and ArdB antirestriction proteins to regulate gene expression in an engineered *E. coli* K-12 MG1655-based biosensor strain. This biosensor strain harbors a *lux*-based reporter system controlled by the AllR-repressed promoter. Although structurally similar, DNA-mimic ArdA proteins interact with AllR differently. Recently described small sArdC and well-known ArdA from the conjugative plasmid R64 appear to bind AllR and open the promoter, while the other tested antirestriction proteins (small sArdN protein and various full-sized ArdA proteins from different sources) have no effect on gene expression under AllR-controlled promoter. Direct binding between ArdA and AllR was experimentally confirmed using pull-down assays with His-tagged ArdA. Our study opens up prospects for the specific use of antirestriction proteins for the regulation of gene expression. Surprisingly, ArdB, a non-DNA-mimic antirestriction protein used initially as a control, was also able to open the promoter, apparently through nonspecific interaction with DNA. We verified this effect with a distant ArdB homolog from a rhizobacterium, which was also able to open the promoter.

## 1. Introduction

Restriction-modification systems (RM) consist of restriction endonucleases and DNA methyltransferase subunits. These systems provide defense against mobile genetic elements by recognizing and cleaving non-methylated foreign DNA, thereby preventing lytic infection by phages, and establishment or genomic integration by plasmids and conjugative elements [1]. To counteract this defense, many plasmids and conjugative elements encode ArdA proteins that mimic B-form DNA in their structure and surface charge. Proteins from the ArdA family inhibit Type I RM enzymes (RMI) by direct binding to the restriction complexes and therefore prevent degradation of mobile genetic element DNA [2]. This protective mechanism facilitates successful horizontal transfer between bacteria, a process that facilitates the rapid spread of beneficial features such as antibiotic resistance, virulence factors, and novel metabolic capabilities [3].

Our recent studies demonstrated that the DNA-mimicry function of ArdA proteins is highly specific towards their RM targets, and we suggested this could be achieved via imitation of specific DNA sequences. Our experiments revealed significant differences in the antirestriction activity of different ArdA proteins against RMI systems, which vary exclusively in their recognition sites [4].

Recently, using transcriptome analysis, we showed that chromosomally encoded ArdA from *Bifidobacterium bifidum* and ArdA from pKM101 oppositely regulate some operons, like the lac operon, involved in lactose catabolism [5]. This study leads to two conclusions. First, the *ardA* genes can be encoded in bacterial chromosomes. Second, when chromosomally encoded, they likely serve a beneficial function for the host—either protection against the host’s own RMs or regulation of gene expression. Given that bacterial cells encode multiple DNA-recognition enzymes such as transcriptional regulators, and considering the high-level expression of DNA mimic proteins, including ArdA, it is plausible that these proteins have additional targets and modulate host metabolism. Thus, chromosomal ArdA proteins may not be an evolutionary atavism but could be involved in bacterial self-regulatory mechanisms.

The discovery of unusually small chromosomal *ardA* genes (*sardN* and *sardC*) [6] raises intriguing questions about their specific mechanisms of action and their potential for distinct regulatory functions compared to their larger relatives.

To elaborate on previous observations that suggested a modulatory role for ArdA DNA mimic proteins in transcription regulation, and to determine whether truncated ArdA variants retain this activity, we constructed a biosensor-based system [7,8] in which the *luxCDABE* genes from *Photorhabdus luminescens* were placed under the control of an inducible promoter *gcl*_P_. To reduce the lux operon copy number, the transcriptional fusion P*gcl*::*luxCDABE* was integrated into the chromosome of *E. coli* MG1655 using the genome editing system developed by D. Bubnov [9].

Using this biosensor setup, we compared the ability of two well-known ArdA proteins from conjugative plasmids R64 and pKM101 and two small sArdC and sArdN proteins to modulate AllR-regulated gene expression in *E. coli*. The regulatory system of the allantoin regulon repressed by AllR was first described in the study [10]. Using transcriptome analysis, we demonstrated [5] that the *ardA* genes could regulate transcription, with evidence indicating that the chromosome-derived *ardA* gene from the *Bifidobacterium bifidum* chromosome often acts in an opposite manner compared to the plasmid-derived gene from pKM101. This effect was also observed for the mRNA of the *gcl* genes belonging to the AllR regulon.

Surprisingly, ArdB, a non-DNA-mimic antirestriction protein used as a control, was also able to open the *gcl* promoter, apparently through nonspecific interaction with DNA.

## 2. Results

### 2.1. Modulation of Gene Expression by Antirestriction Proteins

Previous work revealed that ArdA from plasmid pKM101 and ArdA from the *B. bifidum* chromosome could modulate bacterial gene expression and demonstrated opposite effects on *gclD* and *gclE* genes [5]. We decided to extend these observations and investigate a range of ArdA proteins from different sources, including recently identified truncated sArdN/C, for their ability to regulate the *gcl_p_* promoter.

**Figure 1:**
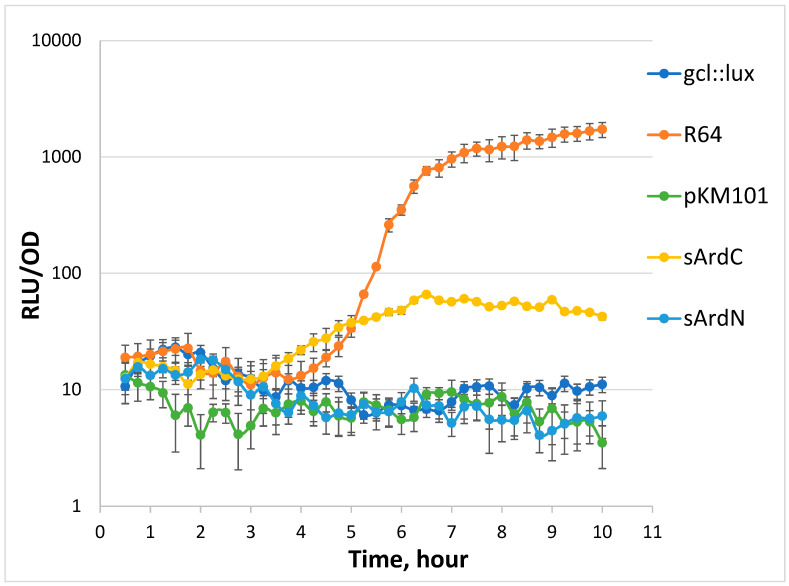
Dynamics of RLU/OD_600_ values of biosensor *E. coli* MG1655 *gcl::lux* strains in the presence of different plasmids, carrying antirestriction proteins, after induction of *ardA* genes. A—*ardA_R64* and *ardA_pKM101* under control of *L-araBAD* promoter after arabinose induction; B—*sArdC* and *sArdN* under control of LuxR2-regulated promoter after temperature induction.

We constructed a biosensor, encoding the *lux* operon under the control of the *gcl_p_* promoter, and tested the ability of eight *ardA* genes from different sources to modulate *lux* operon expression. We found that *ardA* from conjugative plasmid R64 (*ardA_R64)* and truncated *sardA* from *L. cremoris* (*sardC* gene) open the *gcl* promoter, while the other tested *ardA* genes (*ardA* from *B. bifidum* chromosome, *ardA* from *L. lactis* chromosome, *ardA* from *B. longum* chromosome, *ardA* from *P. plecoglossicida* transposone, *ardA* gene from *Arthrobacter* sp. chromosome, *ardA* gene from conjugative plasmid pKM101 (*ardA_pKM101*), *ardA* from transposone Tn916, and *sardA* gene from *C. pilbarense* chromosome (*sardN* gene) did not affect *gcl*-dependent expression. Figure 1 demonstrates the dynamics of the *lux* operon expression after induction of ArdA from conjugative plasmid R64 and *sArdC* from *L. cremoris* on the *gcl* promoter, while other ArdA variants had no effect on luminescence and are omitted.

**Figure 2:**
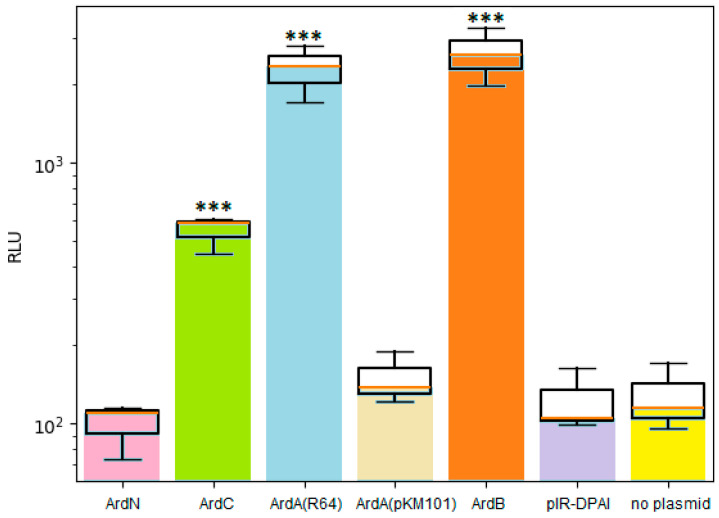
Highest corrected luminescence levels normalized by OD 600 of biosensor *E. coli* MG1655 *gcl::lux* strains in the presence of different pIR-DPAl-based plasmids, carrying antirestriction genes. As negative controls, MG1655 *gcl::lux*—pIR-DPAl and *E. coli* MG1655 *gcl::lux* cells were used. The results of 5 independent replicates are presented. *** denotes statistically significant differences between the indicated bar and the “no plasmid” variant (ANOVA with *t*-test, *p* < 0.005).

For confirmation and quantification of the observed effects, we collected end-point luminescence data from five independent replicates for chosen genes: *ardA_R64*, *ardA_pKM101*, *sardC*, and *sardN*. ArdB_R64, which is a non-DNA-mimic antirestriction protein, was included as a negative control. Another control strain harbored the empty pIR-DPAl vector and exhibited only basal levels of luminescence, consistent with AllR-mediated repression of the *gcl* promoter (Figure 2).

Among full-length ArdA proteins, only ArdA_R64 induced luminescence, whereas ArdA from pKM101 did not elicit any detectable increase in luminescence, indicating the lack of activity. Intriguingly, truncated sArdN and sArdC variants exhibited markedly different behaviors: sArdC induced robust luminescence, while sArdN failed to produce any detectable signal. Overall, these findings confirm that ArdA proteins, including recently discovered truncated sArdC, can modulate gene expression by DNA mimicry.

### 2.2. Modulation of Gene Expression by ArdB Proteins

**Figure 3:**
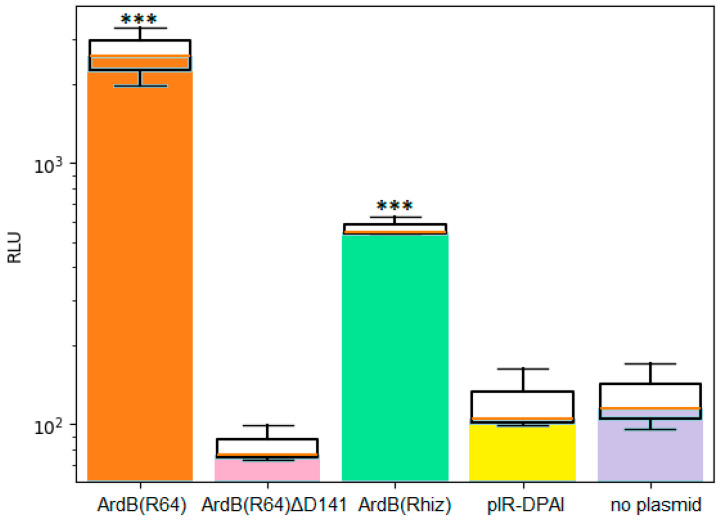
End-point luminescence levels normalized by OD 600 of biosensor *E. coli* MG1655 *gcl::lux* strains in the presence of different pIR-DPAl-based plasmids, carrying antirestriction genes. As negative controls, MG1655 *gcl::lux*—pIR-DPAl and *E. coli* MG1655 *gcl::lux* cells were used. The results of 5 independent replicates are presented. *** denotes statistically significant differences between the indicated bar and the “no plasmid” variant (ANOVA with *t*-test, *p* < 0.005).

Surprisingly, ArdB protein, initially included as a negative control, which was not expected to regulate the promoter due to its lack of DNA-mimicking properties, also activated the *gcl_P_* promoter almost to the same extent as ArdA from the R64 plasmid. Previously, we identified that ArdB has its own DNA-binding activity [11], and therefore potentially may directly interact with the promoter region or could indirectly influence transcription through an alternative mechanism. To confirm that ArdB’s DNA-binding activity is required to modulate the *gcl_P_* promoter, we used ArdB mutant ΔD141, lacking both antirestriction [12] and DNA-binding [11] activities. Notably, ArdBΔD141 did not activate the *gcl_P_* promoter (Figure 3).

To verify the universality of this transcription modulation effect for ArdB family proteins, we investigated a distant homolog of ArdB from the rhizobacterium *S. zoogloeoides* (ArdB_Rhiz, 41% protein identity to ArdB_R64). We found that ArdB_Rhiz activates the *gcl_P_* promoter, albeit less efficiently compared to ArdB(R64) (Figure 3). This can be explained by the fact that ArdB(R64) resides in a more native environment within MG1655 cells compared to the rhizobacterial ArdB_Rhiz, which may lack certain additional factors necessary for non-specific DNA binding or proper folding. The problematic expression/poor folding of rhizobacterial proteins in *E. coli* has been well documented in the literature [13,14,15].

### 2.3. Modeling ArdA-AllR Interaction

Since the interaction between ArdA and AllR/*gclA* remains unexplained so far, we focused on modeling interactions between AllR repressor and DNA-mimicking antirestriction proteins using AlphaFold3.

**Figure 4:**
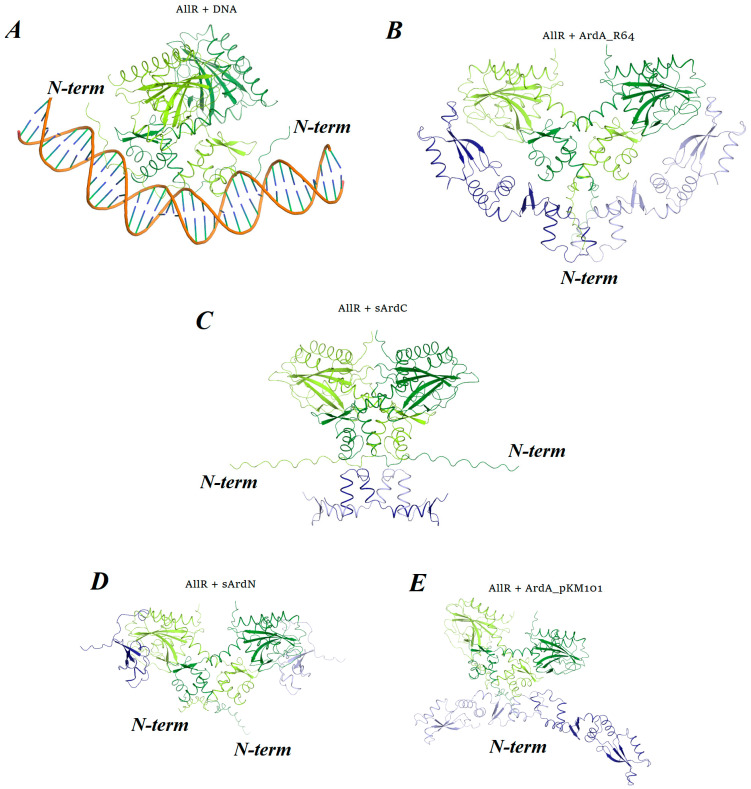
AF3 models of complexes between the dimeric transcriptional regulator AllR with DNA and DNA-mimicking proteins. **N**—DNA-binding N-terminal end of protein AllR. (**A**)—Interaction with DNA (ipTM = 0.49; pTM = 0.5), (**B**)—Interaction with ArdA(R64) dimer (ipTM = 0.27; pTM = 0.35), (**C**)—Interaction with sArdC dimer (ipTM = 0.35; pTM = 0.4), (**D**)—Interaction with sArdN dimer (ipTM = 0.32; pTM = 0.39), (**E**)—Interaction with ArdA(pKM101) dimer (ipTM = 0.26; pTM = 0.33).

Figure 4 demonstrates the model of the AllR dimer [16] interacting with target DNA (AAATCTAGTTTTGGAAAAATATTCCAACTTTTGTATT—AllR recognition site [10]) and with dimers of DNA-mimic ArdA proteins: sArdN, sArdC, ArdA(pKM101), ArdA(R64).

Figure 4A demonstrates that AllR binds to DNA via its N-terminus, which corresponds to a generally accepted model [16]. Apparently, the mechanism of AllR competitive inhibition by DNA-mimic proteins could be realistic, considering that ArdA occupies a DNA-binding interface. AllR forms plausible complexes with ArdA(R64) and sArdC (Figure 4B,C) but likely does not interact with ArdA(pKM101) or sArdN, which lacked transcription modulation activity (Figure 4D,E). However, the ipTM and pTM scores of the modeled complexes were quite low; therefore, we decided to demonstrate direct interaction between ArdA_R64 and AllR in vivo.

### 2.4. ArdA Directly Interacts with AllR, While ArdB Binds to the gcl_P_ Promoter

Native chromosomal expression of AllR is quite low (data not presented). Therefore, to detect AllR:ArdA complexes, we transcriptionally coupled *ardA* from R64 or from pKM101, fused with a 6xHis-tag with the *allR* gene, driving their co-expression by a temperature-inducible promoter in the pIR-DPAl expression vector [17].

**Figure 5:**
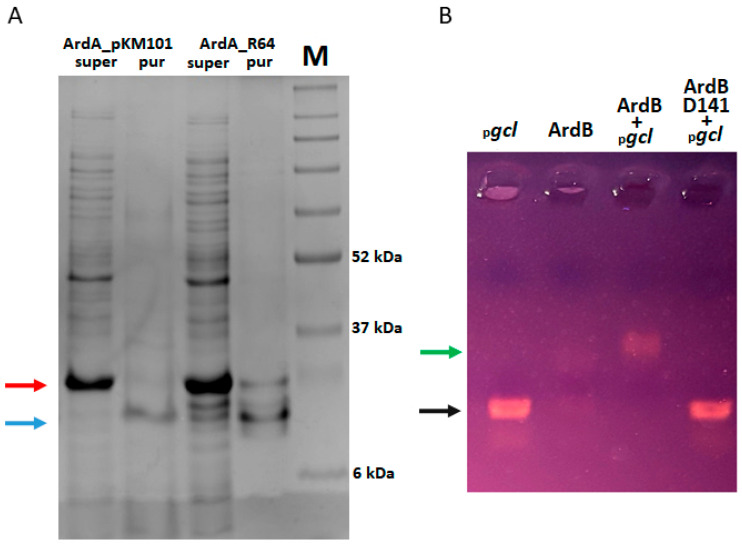
(**A**)—SDS-PAGE of ArdA-His pull-down performed in conditions of co-expression with AllR. AllR is marked with a red arrow, ArdA_pKM101 or ArdA_R64—blue arrow. Super—supernatant of lysed *E. coli* MG16155 pIR-DPAl-AllR-ArdA-pKM101 or pIR-DPAl-AllR-ArdA-R64 cells; pur—purified ArdA_pKM101 or ArdA_R64. M—RAV10 molecular weight marker (Biolabmix, Novosibirsk, Russia). (**B**)—1% agarose gel of PCR product containing the *gcl_P_* promoter sequence binding to ArdB in a 0.5% formaldehyde. Black arrow marks 350 bp _p_*gcl* DNA fragment. Green arrow—retarded band.

We purified ArdA_R64 and ArdA_pKM101 using nickel-affinity chromatography and investigated binding partners via SDS-PAGE. Figure 5A demonstrates that AllR does not co-purify with ArdA_pKM101 protein, while MALDI-TOF analysis of the proteins purified in a pull-down with ArdA_R64 detects AllR among the major binding partners.

Fraction analysis unequivocally shows co-isolation of AllR with ArdA_R64, but not with ArdA_pKM101 (Figure S1).

To confirm that, in contrast to ArdA, ArdB controls *gcl_P_* through direct protein-DNA binding, we performed a gel shift assay. Previously, we demonstrated a non-specific interaction of the ArdB protein with DNA [11]. We purified ArdB_R64 and its mutant form ArdBΔD14, lacking non-specific DNA binding activity, and incubated them with a PCR product containing the *gcl_P_* promoter sequence in vitro using 0.5% formaldehyde as a cross-linking agent [11]. The results are presented in Figure 5B. The DNA-associated protein ArdB forms a complex that is retarded in the agarose gel compared to the unbound DNA, whereas the mutant ArdBΔD141 protein does not bind DNA and therefore does not retard its migration through the gel.

## 3. Discussion

In previous work [5], we identified chromosomally encoded variants of DNA-mimic ArdA proteins. Using transcriptomic analysis, we showed that two *ardA* genes (the chromosomal one from *B. bifidum* and the plasmid one from conjugative plasmid pKM101) are able to regulate gene expression in *E. coli.* Here, we directly demonstrated that some ArdA variants could modulate the expression of the *lux* operon placed under control of the *gcl_P_* promoter regulated by AllR protein. We showed that even truncated ArdA proteins (such as sArdC) can specifically affect the activity of transcription regulators. Although we cannot completely remove indirect effects of heterologous protein expression, such as cellular stress or metabolic perturbations, the growth curves revealed no substantial differences in OD600 dynamics between strains expressing ArdA/ArdB and control strains. This suggests that the observed changes in luminescence are unlikely to be solely explained by general growth defects or global stress responses.

The data shown in Figure 3 suggest that antirestriction proteins of the ArdB family may also participate in the regulation of some genes’ transcription despite their lack of DNA-mimicking properties [18]. It has been previously shown [11] that ArdB could bind to DNA, and this effect depends on a C-terminal glutamate residue [12]. These findings could indicate that ArdB competes with the transcriptional regulator AllR at certain sites on DNA, and EMSA analysis confirms direct interaction of ArdB with the *gcl_P_* promoter. This effect was rather unexpected and requires further investigation to understand how transcription regulation occurs in the presence of ArdB within cells.

The situation is clearer with DNA-mimicking antirestriction proteins. Protein–protein interaction modeling, illustrated in Figure 4, demonstrates that AllR forms a plausible complex with ArdA(R64) and sArdC. In contrast, AllR does not form a complex with ArdA(pKM101) and sArdN. According to AlphaFold predictions (Figure 4), AllR binds to DNA via its N-terminus, which is consistent with the generally accepted view [16]. While AlphaFold3 provides valuable structural insights, the low ipTM and pTM scores (0.26–0.35) for ArdA-AllR complexes suggest that these predictions should be interpreted with caution. Therefore, we experimentally validated the interaction using pull-down assays, which confirmed complex formation only for ArdA_R64, but not for ArdA_pKM101. The mechanism of derepression of AllR-regulated promoters by competitive inhibition with DNA mimic proteins appears to be quite plausible.

This study opens up broad prospects for the precise regulation of cellular processes using DNA mimics, including very small “almost-peptide” molecules such as sArdC.

## 4. Materials and Methods

### 4.1. Bacterial Strains and Plasmids

*Escherichia coli* MG1655 (K-12 F-lambda-*ilvG*-*rfb*-50 *rph*-1), TG1 (K-12 glnV44 thi-1 Δ(lac-proAB) Δ(mcrB-hsdSM)5(rK–mK–) F′ [traD36 proAB+ lacIq lacZΔM15]), BW25113 (K-12 F-lambda-*ilvG*-*rfb*-50 *rph*-1, EcoKI HsdR-), and AB1157 (K-12 F-lambda-EcoKI+ StrR) were used as host strains. The biosensor strain was engineered to contain the Lux luminescence gene cassette under the control of the *gcl* promoter, which is normally repressed by the AllR repressor [10].

A collection of 8 *ardA* genes from different sources was cloned into a p15ara vector [19] under the *L-araBAD* promoter using NdeI and PstI restriction sites. Oligonucleotides for making the new plasmids are presented in Table 1.

The pIR-DPAl expression vector was used [17] as the backbone for constructing plasmids carrying antirestriction genes (*ardA*). Plasmids expressing the *ardA* gene from the R64 conjugative plasmid were constructed previously [17] based on the same pIR-DPAl expression vector.

### 4.2. Biosensor Construction

To explore gene expression, we created a model system based on *E. coli* MG1655 cells, which were engineered to carry a Lux luminescence reporter gene cassette under the control of the *gcl* (glyoxylate) promoter. The *gcl* promoter is tightly regulated by the AllR repressor, which is normally bound to the promoter region, thus preventing transcription of *gcl* genes [10]. These biosensor cells were named *E. coli* MG1655 *gcl::lux*. The strain was constructed using λ Red recombineering [9,21]. The PCR product of the *gcl*_P_ promoter was obtained using primers:

PgclinsF 5′-ttatgacaacttgacggctacatcattcactttttcttcacaaccggcactgtctgtcgcatcccgctc-3′

PgclinsR 5′-atttgccccaacagttgcgcagcctgaatggcgcgagctcggtacccggctacctctatttattggaaaattt-3′

Chromosomal DNA of the *E. coli* MG1655 strain was used as a matrix. Next, through integrative transformation of strain *E. coli* B2095 (*E. coli* MG1655 *ΔaraBAD::luxCDABE ΔaraC::PH207-cI-hok-neo* [9]) using the PCR product, we obtained the biosensor strain MG1655 *Δ*[*araC-araBAD*]*::*P*_gcl_-luxCDABE*. The construct was verified by sequencing using the following primers:

5047araCchrF 5′-agccgtcaattgtctgattcg-3′

4574luxCchrR 5′-aatgttatgcaaccgtaattcg-3′

### 4.3. Design of Experiment and Measurement of Luminescence

We introduced plasmids expressing various ArdA and ArdB proteins into the engineered *E. coli* cells. To investigate the effect of different ArdA proteins on Lux operon expression, *E. coli* MG1655 gcl::lux was transformed with the constructed plasmids with one of the *ardA* genes under control of *L-araBAD* or LuxR2- regulated promoter. We also tested two plasmids with ArdB proteins, which are also antirestriction proteins, but their mechanism is not based on DNA-mimicry.

We first performed dynamic luminescence assays. A single colony of the assayed strain was inoculated into 5 mL of LB medium supplemented with appropriate antibiotics. The overnight culture was diluted to an initial OD 600 of 0.004 with fresh LB medium supplemented with or without 2 mM L-arabinose as an inducer (for p15ara-based plasmids). pIR-DPAl-based plasmids were induced by a temperature downshift as described [17].

Two hundred microliters of the culture were added to each well of a 96-well plate (black-walled, transparent flat bottom; cat. #665096 Greiner Bio-One, Frickenhausen, Germany). The outer wells were not used to avoid edge effects. A well containing a sterile medium was used as a blank. The plates were incubated at 30 °C (or 22 °C for pIR-DPAl-based plasmids) with double-orbital shaking at 600 rpm using a CLARIOstar Plus luminometer (BMG Labtech, Ortenberg, Germany); OD 600 and luminescence were measured every 15 min. No luminescence emission filter was used. The photomultiplier gain was automatically controlled using an enhanced dynamic range function. The measured values were normalized to a 1 s accumulation time. The acquired data were analyzed using MARS version 5.13software. Blank values were subtracted from the raw OD 600 and relative luminescence units (RLU) values. The corrected RLU reads at each time point were divided by the corresponding OD 600 values to normalize the RLU per cell mass for each well. The average RLU/OD 600 values and standard deviations were calculated and plotted against the OD 600.

In order to collect more data for statistics, we performed some additional experiments with representative pIR-DPAl-based strains. The transformed strains were grown overnight in LB broth supplemented with kanamycin, then diluted 1:100 into fresh medium and grown further to mid-log phase. Then, the protein synthesis induction was performed by placing tubes at 22 °C on a tube shaker for 4 h. After that, measurements of luminescence were performed by mixing 200 μL of cell suspension with 0.1% nonanal in a capless tube. Luminescence measurements were performed using a Biotox-7BM luminometer (BioPhysTech, Dolgoprudny, Russia) every half an hour for 16 h. OD 600 was also measured to normalize RLU values. Note: all strains were tested using phage plaque assays to verify antirestriction protein functionality, and SDS-PAGE was used to verify protein expression during luminescence experiments.

### 4.4. Modeling

Protein–protein and protein-DNA interaction models were generated using AlphaFold3 (AlphaFold Server, https://alphafoldserver.com accessed on 10 April 2025). Input sequences (are presented in Table S1) for AllR, ArdA_R64, ArdA_pKM101, sArdC, and sArdN were submitted with default parameters. For protein-DNA modeling, the AllR recognition sequence (5′-AAATCTAGTTTTGGAAAAATATTCCAACTTTTGTATT-3′) was included. Output models were visualized using PyMOL version 2.5.5. (Schrödinger, Inc., New York, NY, USA). Predicted interaction scores (ipTM and pTM) were recorded as reported by AlphaFold3.

### 4.5. ArdA-AllR Co-Purification

To verify ArdA interaction with AllR, we designed two plasmids driving superexpression of the transcriptionally coupled *ardA* and *allR* genes under a thermoinducible promoter. We used chromosomal DNA of *E. coli* MG1655 as a matrix for *allR* gene amplification and the following oligonucleotides:

G_AllR_f 3′-aacattattttaaatatattaataaggaggtatcatatgacggaagttagacggcgcggcaggccag-5′

G_AllR_r 3′-ccagaatggtggtgatggtgatgcatatgatacctcctatttattatggatgtgctttcagtcccaacg-5′;

The PCR product was ligated with pIR-DPAl-ArdA-R64 or pIR-DPAl-ArdA-pKM101, digested with NdeI. As a result, we obtained two plasmids driving superexpression of the *ardA* and *allR* genes under a thermoinducible promoter pIR-DPAl-AllR-ArdA-R64 or pIR-DPAl-AllR-ArdA-pKM101. Only the ArdA protein, but not AllR, had a 6x-His tag.

*E. coli* MG1655 pIR-DPAl-AllR-ArdA-R64 or pIR-DPAl-AllR-ArdA-pKM101 cells were incubated overnight in LB at 22 °C to induce the thermoinducible promoter. Cells were collected by centrifugation and resuspended in 20 volumes of 100 mM Tris-HCl (pH 8.0). After that, cells were lysed using a microfluidizer, and the supernatant was used for Ni-Nta (Abisense, Moscow, Russia) purification according to the manufacturer’s recommendations.

Protein-containing fractions were concentrated using 3 kDa Amicon centrifugal filter units (Merk) and analyzed by SDS-PAGE. The identity of protein bands was determined by matrix-assisted laser desorption/ionization time-of-flight (MALDI-TOF) mass spectrometry. Samples were prepared with Trypsin Gold (Promega) in accordance with the manufacturer’s instructions. Mass spectra were obtained using the rapifleX system (Bruker).

### 4.6. Gel Shift Assay

A 350 bp _p_*gcl* DNA fragment was obtained using a PCR reaction with oligonucleotides Pgcl_BamH_dir 3′-gccggatccgtagtgttgttgccgctatctccatct-5′ and Pgcl_Kpn_rev 3′-gaaggtaccataccgccgtgcttacgcatcgctgag-5′ and *E. coli* MG16155 chromosome as a template.

6x-His-tagged ArdB proteins were isolated using Ni-Nta (Abisense, Russia) purification followed by gel filtration (Superdex 200, GE Healthcare Chicago, USA). A total of 0.6 μg of purified ArdB or ArdBΔD141 was mixed with 0.1 μg of 350 bp *pgcl* DNA fragment and 0.1% formaldehyde for 15 min at room temperature.

## Figures and Tables

**Table 1:** Plasmids used in the present study.

Plasmid	Description	Source
p15ara_1784	Vector p15ara containing *ardA* gene from *Bifidobacterium bifidum* chromosome [5] Oligonucleotides: 1784Nde_dir; CGCCATATGGCGGAAGACGATCTG 1784Pst_rev; GGCCTGCAGGGCGTATGCCGTCGAGCA	This work
p15ara_3194	Vector p15ara containing *ardA* gene from *Lactococcus lactis* chromosome Oligonucleotides: 3194Nde_dir; CGCCATATGGAAACACCAAAAATTTATG 3194Pst_rev; GCCTGCAGTACTTAACCTTTAAATATCTCTGA	This work
p15ara_553	Vector p15ara containing *ardA* gene from *Bifidobacterium longum* chromosome	[20]
p15ara_613	Vector p15ara containing *ardA* gene from *Pseudomonas plecoglossicida* transposone	[20]
p15ara_655	Vector p15ara containing *ardA* gene from *Arthrobacter* sp. chromosome	[20]
p15ara_R64	Vector p15ara containing *ardA* gene from conjugative plasmid R64	[19]
p15ara_pKM101	Vector p15ara containing *ardA* gene from conjugative plasmid pKM101. Oligonucleotides: pKMardF; GCACATATGACTGATATTACGACCCC pKMardR; ATGCTGCAGTCAGCAAGTCATGTTAAATACG	This work
p15ara_Tn916	Vector p15ara containing *ardA* gene from transposone Tn916. Oligonucleotides: Tn916D; GGACATATGGACGATATGCAAGTCTA Tn916Ndrev; GAACTGCAGTCAAGTGTATTCGTCAACCGTAAA	This work
pIR-DPAl	Vector, containing *Aliivibrio logei luxR2* gene and LuxR2-regulated promoter	[17]
pIR-DPAl-ArdA-R64	pIR-DPAl vector with *ardA* gene from conjugative plasmid R64 (ArdA_R64 protein)	[17]
pIR-DPAl-ArdB-R64	pIR-DPAl vector with *ardB* gene from conjugative plasmid R64 (ArdB_R64 protein)	[17]
pIR-DPAl-ArdB_Rhiz	pIR-DPAl vector with *ardB* gene from *Shinella zoogloeoides* chromosome (ArdB_Rhiz).	This work
pIR-DPAl-ArdA-pKM101	pIR-DPAl vector with *ardA* gene from conjugative plasmid pKM101 (ArdA_pKM101 protein). Oligonucleotides: ArdA_pKM101_pIRDPAL_dir 3′- Catcaccatcaccaccatatgactgatattacgacccc-5′ ArdA_pKM101_pIRDPAL_rev; 3′-attgctcagcggtgtcattattcagcaagtcatgttaaatacg-5′	This work
pIR-DPAl-ArdA-1576	pIR-DPAl vector with *ardA* gene from chromosome of *Lactococcus cremoris* (sArdC protein)	[6]
pIR-DPAl-ArdA-8247	pIR-DPAl vector with *ardA* gene from chromosome of *Corynebacterium pilbarense* (sArdN protein)	[6]

## Data Availability

The original contributions presented in this study are included in the article/Supplementary Materials. Further inquiries can be directed to the corresponding author.

## Supplementary Materials

The following supporting information can be downloaded at: https://www.mdpi.com/article/10.3390/ijms27125595/s1.
