# Supplementary material for: DNA-Mimic Antirestriction Proteins ArdA Could Regulate Gene Expression in Escherichia coli
Source: Int J Mol Sci. 2026 Jun 20;27(12):5595. doi: 10.3390/ijms27125595 (PMC13300388; doi:10.3390/ijms27125595)
Supplement: Supplementary file 1 [file ijms-27-05595-s001.zip › ijms-4343388-supplementary.pdf]

Table S1. Protein input sequences for AlphaFold modeling.

Protein	Sequence
**AllR**	MTEVRRRGRPGQAEPVAQKGAQALERGIAILQYLEKSGGSSSVSDISLNLDLPLSTTFRLLKVLQAADFV<br>YQDSQLGWWHIGLGVFNVGAAYIHNRDVLSVAGPFMRRLMLLSGETVNVAIRNGNEAVLIGQLECKS<br>MVRMCAPLGSRLPLHASGAGKALLYPLAEEELMSIILQTGLQQFTPTTLVDMPTLLKDLEQARELGYTV<br>DKEEHVVGLNCIASAIYDDVGSVVAAISISGPSSRLTEDRFVSQGELVRDTARDISTALGLKAHP
**ArdA_pKM101**	MTDITTPSVYVGTYHKYNCGSIAGAWLDLTDFDSSEEFYERCRELHANEADPEFMFQDWEGIPSDMASE<br>CHINWDFINGFKQAREEGNEAAFVAFVDLFNSTDFDLFRDAYMGEAKDEETFAEEYLNDSGLLNEIPES<br>VARYFDIVAYARDLFIGDFSLHDGHVFNMTC
**ArdA_R64**	SVVAPAVYVGTWHKYNCGSIAGRWFDLATFDDERDFFAACRSLHQDEADPELMFQDYEGFPGNMASE<br>CHINWAYVEGFRQARDEGCEEAYRLWVDDTGETDFDTFRDAWWGEADSEEAFAVEFASDTGLLADVP<br>ETVALYFDYEAYARDLFLDSFTFIDGHVFRR
**sArdN**	MDTNNLDSTPRVWIGCLHCYNAGRLVGEWFDAVDADEATLADVHRDAGGSCVGCDELWCFNHENLP<br>VRGEMGPNEAAE
**sArdC**	MEAENDEDLAQELIEQMGGLEVLSIETLQRYFNFSAYGRDLAIGDYSKTSHGYIRDI

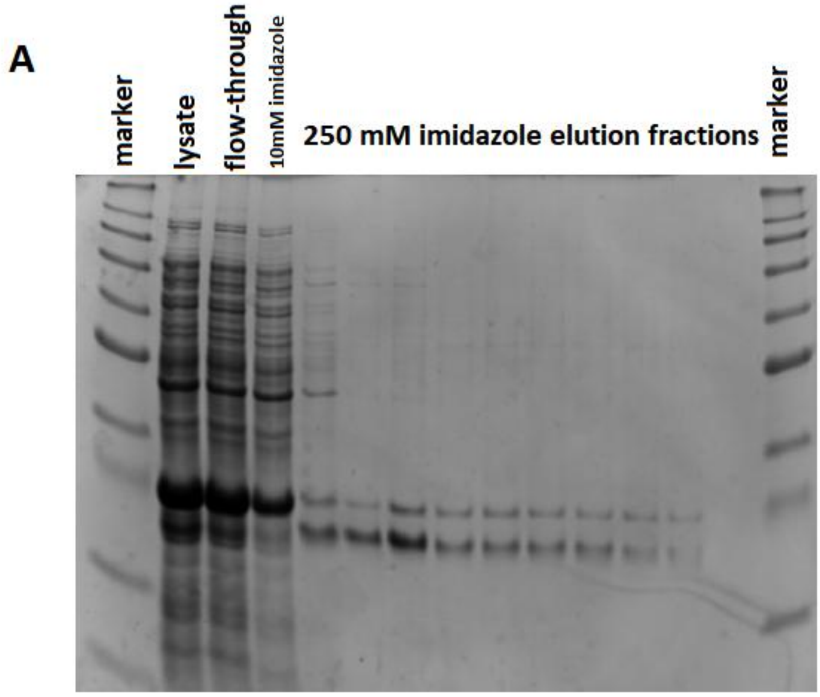

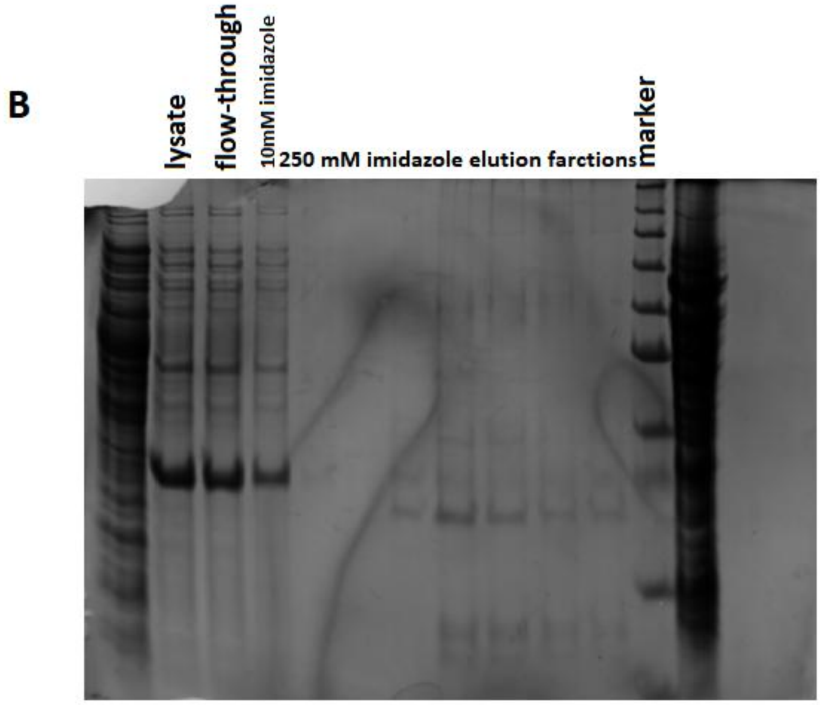

Figure S1. SDS-PAGE of ArdA-His pull-down performed in conditions of co-expression with AllR. A – co-purification with ArdA_R64; B – co-purification with ArdA_pKM101. Fractions are loaded in the order of collection.
